# Effects of breaking up prolonged sitting on vascular endothelial function in adults: a systematic review

**DOI:** 10.3389/fcvm.2026.1780316

**Published:** 2026-09-04

**Authors:** Jun He, Xinyu Xu, Yanan Zhao, Liquan Cao, Zhanpeng Feng

**Affiliations:** 1 Tianjin University of Sport, Tianjin, China; 2 Tianjin Key Laboratory of Sports and Health Integration and Health Promotion, Tianjin, China

**Keywords:** adults, endothelium, interrupted sedentary activity, sedentary behavior, vascular endothelial function

## Abstract

**Objective:**

Sedentary behavior contributes to vascular endothelial dysfunction and elevated cardiovascular disease risk. Because endothelial responses may differ by vascular bed, this review systematically evaluated the effects of breaking up prolonged sitting on adults' vascular endothelial function, focusing on flow-mediated dilation (FMD), blood flow, shear-rate outcomes, and the artery or measurement location reported in each study.

**Methods:**

Based on the PRISMA guidelines, literature searches was conducted in PubMed, Web of Science, CNKI, VIP, and Wanfang databases using English search terms “sedentary, sitting, sedentary behaviour, sitting position, standing, exercise, endothelial function, endothelial dysfunction, endothelium” and Chinese search terms “sedentary, sitting, sedentary behaviour, sitting position, standing, exercise, endothelial function, endothelial dysfunction, endothelium”. The search period spanned from January 2000 to November 2025. The PEDro scale was used to assess the quality of the literature, and a review analysis was conducted on the 24 included articles.

**Results:**

Interrupting prolonged sitting generally improved peripheral hemodynamics, especially lower-limb blood flow and shear-rate responses, whereas FMD responses were heterogeneous and depended partly on the artery examined (e.g., brachial, superficial femoral/femoral, or popliteal artery), participant characteristics, and interruption dose. Across the included studies, frequent 2–3 min lower-limb dynamic or simple resistance breaks every 20–30 min appeared most consistently beneficial for hemodynamic outcomes.

**Conclusion:**

Short-term breaks from prolonged sitting may protect vascular function by improving blood flow and shear-rate profiles, particularly in lower-limb arteries. Because FMD findings are site-specific, brachial, femoral/superficial femoral, and popliteal artery results should be interpreted separately when applying these findings to practice.

**Systematic Review Registration:**

https://www.crd.york.ac.uk/PROSPERO/view/CRD420261332434, PROSPERO CRD420261332434.

## 1 Introduction

The vascular endothelium is a monolayer of cells lining the inner surface of blood vessels. It regulates vascular tone, blood fluidity, inflammation, permeability, and thrombosis through mediators such as nitric oxide (NO), endothelin-1, and prostacyclin. Vascular endothelial dysfunction refers to impaired endothelial regulation, particularly reduced NO-mediated vasodilation, and is considered an early functional abnormality in the development of atherosclerosis and cardiovascular disease (1–5). Flow-mediated dilation (FMD) is commonly used to assess conduit-artery endothelial function: after transient cuff-induced ischemia, the post-occlusion increase in blood flow raises shear stress on the artery wall, and ultrasound is used to quantify the percentage increase in arterial diameter. A lower FMD therefore indicates impaired endothelium-dependent vasodilatory function (5). Shear stress plays a pivotal role in maintaining endothelial function and regulating vasodilation, fibrinolysis, anti-inflammatory, and anticoagulant pathways (6). Importantly, the prognostic evidence is strongest for brachial artery FMD; meta-analytic data indicate that every 1% higher brachial artery FMD is associated with a 13% lower future risk of cardiovascular events (7, 8). This association should not be extrapolated directly to FMD measured in other arteries.

Sedentary behavior, defined as any waking behavior characterized by low energy expenditure (≤1.5 metabolic equivalents) ≤ 1.5 metabolic equivalents of task [METs],while in a sitting, reclining, or lying posture has become increasingly prevalent (9). National surveys in the United States from 2001 to 2016 revealed a rise in daily sedentary time from 7.0 to 8.2 h among adolescents (aged 12–17 years) and 8.8 h among adults. Similarly, data from the China Kadoorie Biobank study indicated that Chinese adults spend an average of 5.0 h per day in sedentary behavior, with urban populations exceeding 6 h. Globally, the World Health Organization estimates that approximately 27.5% of adults fail to meet recommended physical activity guidelines, with sedentary behavior being a major contributing factor (10, 11). Prolonged sedentary behavior adversely affects body composition (12), the cardiovascular system, the musculoskeletal system, glucose metabolism, lipid metabolism, and vascular function (13). Restaino et al. (14). demonstrated that 6 h of sedentary behavior induces lower limb endothelial dysfunction, accompanied by reduced shear rate and FMD. Furthermore, each additional hour of sedentary time beyond 10 h daily increases cardiovascular disease incidence by 8% (15).

Sedentary interruptions refer to non-sedentary behaviors occurring between two periods of sedentary time (16). Saunders et al. (17) conducted a meta-analysis on the acute metabolic and vascular impact of interrupting prolonged sitting, showing that activity breaks can improve vascular outcomes compared with uninterrupted sitting. Subsequent studies by Soto-Rodriguez et al. (2) and Zheng et al. (18) reported increases in FMD, peripheral vascular shear rate, and blood flow after sedentary-break interventions. However, the vascular bed examined varied across studies, including brachial, superficial femoral/femoral, popliteal, carotid, and middle cerebral artery outcomes. Morishima et al. (19) found that leg fidgeting maintained lower-limb endothelial function and improved local shear rate and blood flow, whereas Chandran et al. (20) observed no significant improvement in carotid or superficial femoral artery hemodynamic measures after walking or stair-climbing breaks. Taken together, different sedentary interruption strategies and measurement locations produce varying degrees of vascular response.

Given the ongoing controversy in the existing evidence, further exploration of the effects of sedentary interruptions on adult vascular endothelial function is necessary. The contradictory findings may be attributable to several factors: (1) heterogeneity in intervention modalities and exercise doses across studies, (2) differences in participant characteristics (e.g., age, health status, baseline vascular function), (3) variations in measurement timing and protocols for vascular outcomes, and (4) differences in the artery or vascular bed measured. Unlike previous meta-analyses that pooled heterogeneous data, the present systematic review adopts a narrative synthesis approach to critically appraise the methodological quality and contextual factors of each study, thereby identifying the sources of heterogeneity rather than obscuring them through statistical aggregation. We hypothesize that the effectiveness of sedentary interruption on vascular function is modulated by interruption intensity and frequency, participant health status, and measurement location. Therefore, in accordance with the PRISMA guidelines for evidence-based research (21), this systematic review aims to clarify the impact of sedentary interruption strategies on shear rate, FMD, and blood flow, while explicitly considering the artery examined in each study.

## 2 Materials and methods

### 2.1 Research framework

This study employed a systematic review approach to evaluate existing literature on the effects of sedentary interruption strategies on vascular shear rate, FMD, and blood flow in adults, using the PICO framework (Population, Intervention, Comparison, Outcome). Population - adults aged 18 years and above, of both sexes and any ethnicity, including healthy individuals and those with metabolic or cardiovascular risk factors such as overweight/obesity, type 2 diabetes, hypertension or prehypertension, polycystic ovary syndrome, older age, prolonged occupational sitting, or night-shift work; Intervention - any form of physical activity performed to interrupt prolonged sedentary time, including walking, stair climbing, resistance exercises, heel raises, cycling, and passive/active leg movements; Comparison - uninterrupted prolonged sitting (control condition) or alternative interruption strategies with different frequencies, durations, or modalities; Outcome - primary outcomes: peripheral shear stress, peripheral blood flow, and flow-mediated dilation (FMD), with the artery or measurement location extracted wherever reported; secondary outcomes: pulse wave velocity/arterial stiffness, cerebral blood flow velocity, and other cardiovascular disease (CVD) risk factors. The detailed PICO framework is presented in Table 1.

**Table 1:** PICO framework.

Population	Intervention	Comparison	Outcome
Population: Adults aged 18 years and above, of both sexes, without restriction on ethnicity or nationality, including healthy adults, overweight/obese adults, adults with stage 1 hypertension, adults with type 2 diabetes mellitus, and adults with polycystic ovary syndrome (PCOS). Both Chinese and international populations were included to enhance the generalizability of findings. Age: 18 years and above., of both sexes, without restriction on ethnicity or nationality	Intervention methods: stair sprint interval training; walking with varying intensity followed by rest; heel raises; resistance exercises with rest intervals; intermittent calf raises; intermittent standing or stepping; 1-minute light-intensity half-squat with calf raises; mild leg exercises; isometric leg stretches; intermittent treadmill training;intervention frequency intervention duration	Comparison of sedentary behavior (SED/SIT) with different exercise-rest patterns (e.g., 2WALK vs. 8WALK, walking vs. stair climbing) before vs. after intervention	Peripheral shear stress, peripheral blood flow, peripheral vasodilation function, peripheral pulse wave, cerebral blood flow velocity, and other cardiovascular disease (CVD) risk factors

### 2.2 Literature search

A comprehensive literature search was performed using both English- and Chinese-language databases. The English databases included Web of Science and PubMed, while the Chinese databases included China National Knowledge Infrastructure (CNKI), VIP Database, and Wanfang Data. The search period covered publications from January 2000 to November 2025.

The Chinese search strategy was as follows:([sedentary behavior]OR[sitting posture]OR[sedentary]OR[sitting position]OR[standing]OR[exercise]) AND ([endothelial function]OR[endothelial dysfunction]OR[endothelium]). All Chinese search terms are provided here with their English translations in brackets for clarity.

The English search strategy was as follows:(sedentary OR sitting OR “sedentary behaviour” OR “sitting position” OR standing OR exercise) AND (“endothelial function” OR “endothelial dysfunction” OR endothelium) The search terms were designed to capture all vascular outcomes relevant to sedentary interruption, including hemodynamic indicators (shear stress, blood flow) and endothelial function (FMD). Cerebral blood flow velocity and pulse wave parameters were identified as secondary outcomes during full-text screening, as these measures were reported in several included studies that met the broader vascular function criteria.

### 2.3 Inclusion and exclusion criteria

Inclusion criteria: (1) randomized crossover trials examining the effects of sedentary interruption on vascular function; (2) participants aged 18 years and above, including healthy adults and adults with clearly reported risk characteristics such as overweight/obesity, hypertension or prehypertension, type 2 diabetes, polycystic ovary syndrome, older age, occupational sitting, or night-shift work; (3) interventions involving any type of physical activity as a means to interrupt sedentary time; and (4) outcome measures including at least one vascular function indicator (e.g., peripheral blood flow, shear stress, FMD, pulse wave velocity, central vascular function), with artery or measurement location recorded when available.

Exclusion criteria:①Qualitative studies, systematic reviews/meta-analyses, research protocols, gray literature, and abstracts.②Studies using exercise before continuous sedentary periods as interventions.③Incomplete intervention studies.④Non-peer-reviewed literature or literature unrelated to the topic.

### 2.4 Literature screening and data extraction

Two researchers independently extracted data using a standardized form. Discrepancies were resolved through discussion or consultation with a third researcher. Extracted information included study basics (authors, publication year), country, study design, sample size, age, intervention details, outcome measures related to shear stress, FMD, and blood flow, and the specific artery or measurement location examined in each study whenever this information was reported.

### 2.5 Quality assessment

The PEDro scale was used to assess the methodological quality of the included studies (22). This systematic review was prospectively registered in the PROSPERO international prospective register of systematic reviews (Registration number: CRD42026133243).

## 3 Results

**Figure 1:**
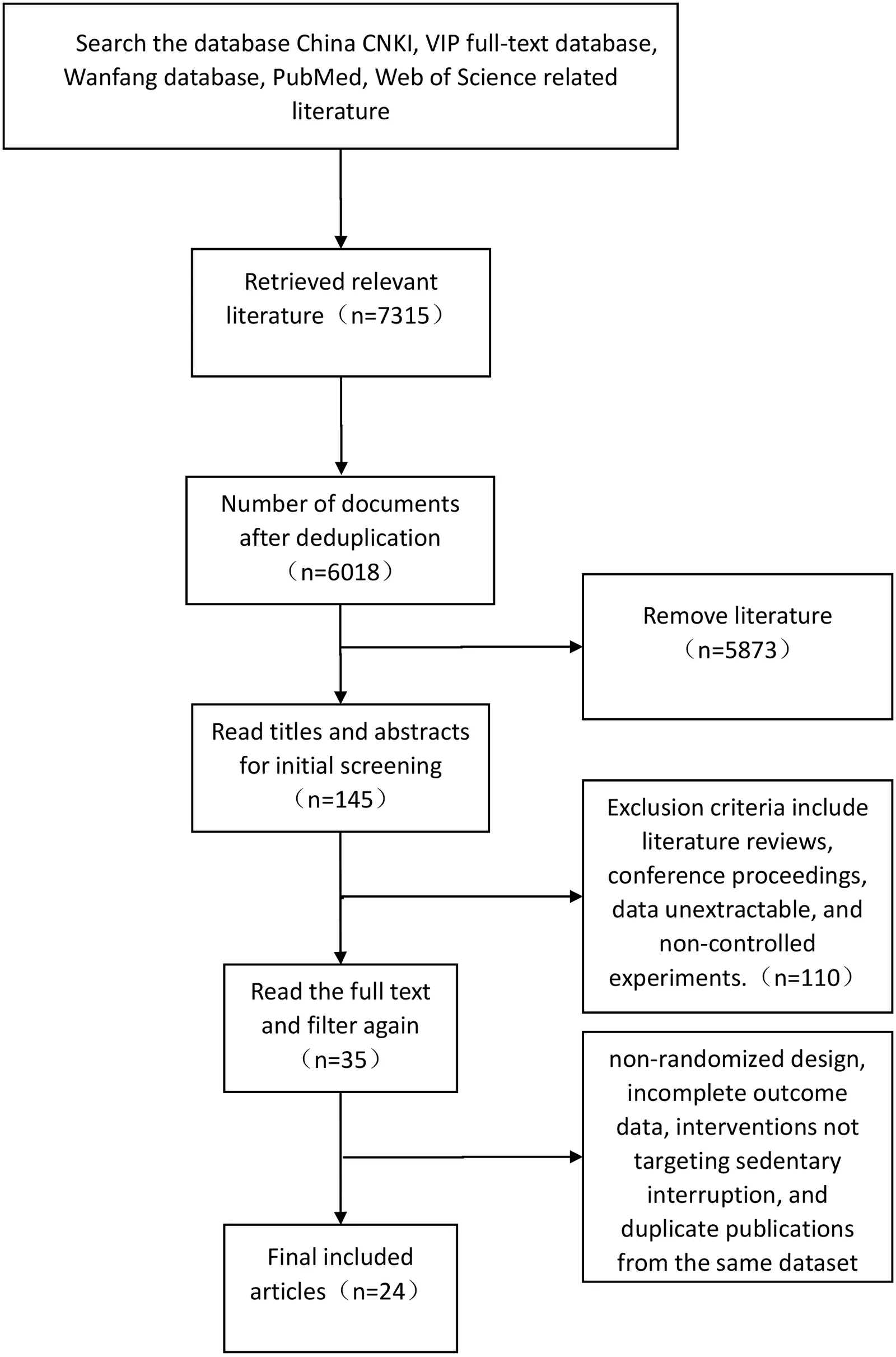
Flowchart of literature screening.

A total of 24 articles were ultimately included (20–46). The study selection process is illustrated in the PRISMA flow diagram (See Figure 1).

### 3.1 Literature quality evaluation

All 24 included studies were rated 7 on the PEDro scale, indicating good methodological quality (Table 2). The PICO framework guiding this review is summarized in Table 1.

**Table 2:** Quality assessment of included studies.

Inclusion criteria	Qualification criteria	random allocation, random assortment	Allocation concealment	baseline similarity	blinded subject	Therapist blind	blinded evaluator	The participant dropout rate is less than 15%.	intention-to-treat analysis	Statistical comparison between groups	report point measurement and variation value	aggregate score
Chandran et al. (20)（2023）	√	√	×	√	×	×	√	√	√	√	√	7
Caldwellet al (24)（2021）	√	√	×	√	×	×	√	√	√	√	√	7
Carter et al. (25)（2018）	√	√	×	√	×	×	√	√	√	√	√	7
Carter et al. ^(^26)（2019）	√	√	×	√	×	×	√	√	√	√	√	7
Carter et al. (27)（2016）	√	√	×	√	×	×	√	√	√	√	√	7
Climie et al. (28)（2018）	√	√	×	√	×	×	√	√	√	√	√	7
Evans et al. (29)（2019）	√	√	×	√	×	×	√	√	√	√	√	7
Hartman et al. (30)（2021）	√	√	×	√	×	×	√	√	√	√	√	7
Kruse et al. (31)（2018）	√	√	×	√	×	×	√	√	√	√	√	7
Horiuchi et al. (32)（2022）	√	√	×	√	×	×	√	√	√	√	√	7
Morishima et al. (33)（2016）	√	√	×	√	×	×	√	√	√	√	√	7
Park et al. (34)（2022）	√	√	×	√	×	×	√	√	√	√	√	7
Peddie et al. (35)（2021）	√	√	×	√	×	×	√	√	√	√	√	7
Perdomo et al. (36)（2019）	√	√	×	√	×	×	√	√	√	√	√	7
Silva et al. (37)（2021）	√	√	×	√	×	×	√	√	√	√	√	7
Taylor et al. (38)（2021）	√	√	×	√	×	×	√	√	√	√	√	7
Taylor et al. (39)（2021）	√	√	×	√	×	×	√	√	√	√	√	7
Thosar et al. (40)（2015）	√	√	×	√	×	×	√	√	√	√	√	7
Wheeler et al. (41)（2019）	√	√	×	√	×	×	√	√	√	√	√	7
Chung et al. (42)（2017）	√	√	×	√	×	×	√	√	√	√	√	7
Lim et al. (43)（2015）	√	√	×	√	×	×	√	√	√	√	√	7
Eguchi et al. (44)（2013）	√	√	×	√	×	×	√	√	√	√	√	7
Murtagh et al. (45)（2005）	√	√	×	√	×	×	√	√	√	√	√	7
Murphy et al. (46)（2002）	√	√	×	√	×	×	√	√	√	√	√	7

### 3.2 Basic characteristics of the included studies

A total of 24 studies were included (20–46), all of which were published in English between 2002 and 2023. These studies employed randomized controlled or randomized crossover designs and comprised a total of 454 participants. The study populations included adults aged 18 years and older, encompassing healthy adults, overweight or obese adults, older adults with increased cardiovascular risk, adults with hypertension or prehypertension, patients with type 2 diabetes mellitus, women with polycystic ovary syndrome, sedentary office workers, and night-shift workers. In this review, increased cardiovascular or metabolic risk was defined according to the original studies' reported characteristics, including elevated BMI/overweight or obesity, hypertension or prehypertension, type 2 diabetes, polycystic ovary syndrome, older age, prolonged occupational sitting, or night-shift work. The basic characteristics of the included studies, control group descriptions, key indicators, and measurement locations are summarized in Table 3.

**Table 3:** Basic characteristics of included literatures.

Inclusion criteria/years of publication	research design	subjects			Intervention Plan		final result
		Sample size (male/female, n)	Mean age in years	healthy condition	intervention group	control group	Key indicators; measurement location/artery
Chandran et al. (20)（2023）	randomized crossover trial	21（17/4）	26.65 ± 2.64	BMI 22.32 ± 2.46，randomized crossover trial	Three types of interventions: 1) 4-hour continuous sitting (SIT); 2) 3-minute light walking (LIT) per hour; 3) 3-minute stair climbing (MIT) per hour	No separate control group was established, with SIT serving as the control.	①② CA/SFA: diameter, velocity, SR, BF
Caldwell et al. (24)（2021）	randomized crossover trial	10（10/0）	24 ± 4	BMI 24 ± 2，healthy male	8.5 h of seated meditation + 1 stair sprint per hour (14-20 s per sprint, total 8 sprints)	8.5 h of uninterrupted sitting	①②③ Femoral/SFA: SR, BF, FMD; cerebrovascular regulation
Carter et al. (25)（2018）	randomized crossover trial	15（10/5）	35.8 ± 10.2	BMI 25.5 ± 3.2，The "healthy office workers"	Two intervention groups: 1) 4-hour sedentary period+2-minute light walking every 30 min (2WALK); 2) 4-hour sedentary period+8-minute light walking every 120 min (8WALK)	4-hour continuous sitting (SIT)	⑤ MCAv: cerebral BF velocity
Carter et al. (26)（2019）	randomized crossover trial	15（10/5）	35.8 ± 10.2	BMI 25.5 ± 3.2，The "healthy office workers"	Two intervention groups: 1) 4-hour sedentary period + 2-minute light walking every 30 min (2WALK); 2) 4-hour sedentary period + 8-minute light walking every 120 min (8WALK)	4-hour continuous sitting (SIT)	①②③ SFA: endothelial function, SR, BF, FMD
Carter et al. (27)（2017）	randomized crossover trial	10（6/4）	27.3 ± 8.1	BMI Not specified, healthy population, high physical activity level	1h26minSit-down exercise + 2-minute aerobics every 20 min (squatting, arm circles, etc.)	1h26min Uninterrupted sitting meditation	①③ Brachial artery: SR, FMD
Climie et al. (28)（2018）	randomized crossover trial	19（11/8）	57 ± 12	BMI 30.6 ± 3.4，Overweight and obesity	5 h of seated meditation + 3 min of simple resistance exercises every 30 min (half squats, heel raises, etc.)	5h Uninterrupted Sitting (SIT)	①②③ Femoral artery: FMD, BF, SR; brachial FMD
Evans et al. (29)（2019）	randomized crossover trial	20（6/14）	21.7 ± 2.5	BMI 25.5 ± 6.1，healthy young adults	3 h of sitting+10 heel raises every 10 min (CALF)	3 h of continuous sitting (CON)	④ Aortic stiffness
Hartman et al. (30)（2021）	randomized crossover trial	24（9/15）	65 ± 5	BMI 29.8 ± 3.9，presence of cardiovascular risk factors (hypertension, overweight, etc.)	16-week sedentary reduction intervention (via mobile health device reminders to increase standing and walking time), including 2 acute interventions: 1) 3-hour continuous sedentary intervention (SIT); 2) 3-hour sedentary + 2-minute light walking every 30 min (BREAKS)	Pre-intervention self-control + 3-hour continuous sitting	②③ SFA FMD; CBFv (transcranial Doppler)
Kruse et al. (31)（2018）	randomized crossover trial	13（10/3）	38 ± 3	BMI 29.7 ± 2，Overweight/obese office workers with prolonged sedentary behavior	Two intervention regimens: 1) 4-hour sedentary + 10-minute standing per hour; 2) 4-hour sedentary + 10-minute desk stepping (moderate intensity)	4 h of uninterrupted sitting	①②③ Popliteal artery: FMD, SR, BF
Horiuchi et al. (32)（2022）	randomized crossover trial	20（10/10）	21 ± 2	BMI 21.5 ± 1.6，healthy young adults	3-hour seated meditation+1-minute light half-squat every 20 min + heel raises (15 repetitions per minute)	3 h of continuous sitting (CON)	④ Lower-limb arterial stiffness/microvascular perfusion
Morishima et al. (33)（2016）	randomized crossover trial	11（7/4）	26 ± 1	BMI 25.0 ± 1.1，healthy young adults	3-hour sitting + intermittent leg tremor on one side（1min/4min，Fidgetingleg）	The other leg remains stationary (Control leg, self-control).	①②③ Popliteal artery: FMD, SR, BF
Park et al. (34)（2022）	randomized crossover trial	14（7/7）	24 ± 2	BMI 24.5 ± 3.7，healthy young adults	Three intervention groups (all subjected to 2.5 h of sedentary posture in a mildly hypercapnic environment with CO₂ = 1500 ppm): 1) No exercise (CON); 2) Passive leg movement (PASS) for 2 min every 30 min; 3) Active leg movement (ACT, 13W load) for 2 min every 30 min.	No additional control group was established, with CON serving as the control.	③④ Brachial and popliteal artery FMD; microvascular function
Peddie et al. (35)（2021）	randomized crossover trial	18（11/7）	23.5 ± 5.0	BMI 23.7 ± 2.6，healthy sedentary adults	Three types of interventions: 1) 6-hour continuous sitting (SIT); 2) 6-hour standing (STAND); 3) 6-hour sitting + 2-minute walking every 30 min (5 km/h, 10% incline)	6-hour continuous sitting (SIT)	①②③ Popliteal artery: FMD, BF, net SR
Perdomo et al. (36)（2019）	randomized crossover trial	24（16/8）	42 ± 12	BMI 31.9 ± 5.0，Overweight/obesity, Pre-1 stage hypertension	7h40min simulated workday: alternating between standing and sitting (SS) every 30 min	7h40min Sustained Isometric Training (SIT)	④⑤ MCAv/pulsatility
Silva et al. (37)（2021）	randomized crossover trial	17（6/11）	29 ± 10	BMI 25.1 ± 5.1，healthy sedentary adults	Three types of interventions: 1) 3-hour sedentary + 2-minute isometric knee extension exercise every 30 min (30% of maximal voluntary contraction); 2) 3-hour sedentary + 2-minute light walking every 30 min; 3) 3-hour continuous sedentary (CON)	3h continuous sitting (CON)	①②③ Vascular function; artery site not stated in abstract
Taylor et al. (38)（2021）	randomized crossover trial	24（13/11）	61.5 ± 7.8	BMI 32.6 ± 3.5，patients with type 2 diabetes mellitus	7-hour sitting + 2 types of interventions: 1) Simple Resistance Activity (SRA3) for 3 min every 30 min; 2) Simple Resistance Activity (SRA6) for 6 min every 60 min	7h Uninterrupted Sitting (SIT)	①②③ SFA: SR, BF, FMD
Taylor et al. (39)（2021）	randomized crossover trial	13（0/13）	32.2 ± 6.3	BMI 30.2 ± 5.3，Women with polycystic ovary syndrome (PCOS)	3.5 h of seated meditation + 3-minute simple resistance exercises every 30 min (half squats, heel raises, etc.)	3.5h Uninterrupted Sitting (SIT)	①②③ SFA: SR, BF, FMD
Thosar et al. (40)（2015）	randomized crossover trial	12（12/0）	24.2 ± 4.2	BMI 23.7 ± 3.4，healthy sedentary males	3-hour sitting session + 5-minute light walking (2 mph) every 30/90/150 min	3h Uninterrupted Sitting (SIT)	①③ Femoral artery: FMD, SR
Wheeler et al. (41)（2019）	randomized crossover trial	12（10/2）	70 ± 7	BMI 30.4 ± 4.3，overweight/obese elderly	Three types of interventions: 1) 8-hour sustained sitting (SIT); 2) 1-hour sitting + 30-minute moderate-intensity walking + 6.5-hour sustained sitting (EX + SIT); 3) 1-hour sitting + 30-minute moderate-intensity walking + 6.5-hour sitting (3-minute light walking every 30 min) (EX + BR)	8h continuous sitting (SIT)	⑤ MCAv/cerebrovascular hemodynamics
Chung et al. (42)（2017）	randomized crossover trial	36（0/36）	48.9 ± 4.6	BMI 25.1 ± 2.7，middle-aged obese women	Three intervention groups: 1) 3 days/week, 1 session/day of 30-minute treadmill exercise (single group); 2) 3 days/week, 3 sessions/day of 10-minute treadmill exercise (multiple groups); 3) No exercise (control group).	using the non-exercise group as control	⑥ CVD risk biomarkers; no artery-specific vascular measurement
Lim et al. (43)（2015）	randomized crossover trial	30（30/0）	57.6 ± 1.8	BMI 23.3 ± 1.9，Male night shift workers	Two intervention groups: 1) 10-week, 3 days/week, 3 times/day, 10-minute brisk walking (exercise group); 2) no exercise (control group)	using the non-exercise group as control	⑥ CVD risk biomarkers; no artery-specific vascular measurement
Eguchi et al. (44)（2013）	randomized crossover trial	23（23/0）	43.9 ± 11.6	BMI 25.8 ± 3.2，Sedentary workers	Two intervention groups: 1) 20 weeks, 3 days/week, 1 session/day of 30-minute power cycling exercise (long-term group); 2) 20 weeks, 3 days/week, 3 sessions/day of 10-minute power cycling exercise (short-term group).	No additional control group, with cross-over design between the two groups	⑥ CVD risk factors; no artery-specific vascular measurement
Murtagh et al. (45)（2005）	randomized crossover trial	32（15/17）	45.7 ± 9.4	BMI＜30kg/m²，healthy sedentary adults	Two intervention groups: 1) 20 weeks, 3 days/week, 1 session/day of 30-minute power cycling exercise (long-term group); 2) 20 weeks, 3 days/week, 3 sessions/day of 10-minute power cycling exercise (short-term group).	No additional control group, with cross-over design between the two groups	⑥ CVD risk factors; no artery-specific vascular measurement
Murphy et al. (46)（2002）	randomized crossover trial	21（7/14）	44.5 ± 6.1	BMI 26.8 ± 3.5，healthy sedentary adults	Two intervention groups: 1) 5 days/week, 1 time/day for 30 min of brisk walking (long-term group); 2) 5 days/week, 3 times/day for 10 min of brisk walking (short-term group)	No additional control group, with cross-over design between the two groups	⑥ CVD risk factors; no artery-specific vascular measurement

Table notes: ①Peripheral shear stress ②Peripheral blood flow ③Peripheral FMD ④Peripheral pulse wave ⑤Cerebral blood flow velocity ⑥Other cardiovascular disease (CVD) risk factors Abbreviations added for measurement location: CA, carotid artery; SFA, superficial femoral artery; SR, shear rate; BF, blood flow; FMD, flow-mediated dilation; MCAv, middle cerebral artery blood flow velocity; CBFv, cerebral blood flow velocity.

### 3.3 Exercise programs

Interventions were categorized into three types based on exercise characteristics, muscle group involvement, and physiological load.

(1) Dynamic lower-limb activities, including walking, stair climbing, and brisk treadmill walking, which rely on rhythmic skeletal muscle pump activity to enhance lower-limb blood flow;
(2) Resistance or resistance-like bodyweight exercises, such as half squats, heel raises, isometric knee extensions, and aerobic-style movements, which induce pronounced transient hemodynamic responses through short-duration, high-tension muscular stimulation;
(3) Passive or low-amplitude localized movements, including intermittent leg fidgeting and desk-based pedaling, which, despite their relatively low intensity, are capable of generating measurable gradients in lower-limb blood flow velocity.

Specifically, the interventions included light walking (Chandran et al. (20), 2023; Carter et al. (25), 2018; Carter et al. (26), 2019; Hartman et al. (30), 2021; Peddie et al. (35), 2021; Silva et al. (37), 2021; Thosar et al. (40), 2015; Wheeler et al. (41), 2019), brisk treadmill walking (Chung et al. (42), 2017; Lim et al. (43), 2015; Murphy et al. (46), 2002), stair climbing [Chandran et al. (20), 2023; Caldwell et al. (24), 2021], resistance training (Climie et al. (28), 2018; Taylor et al. (38), 2021; Taylor et al. (39), 2021), aerobic-style exercise (Carter et al. (27), 2017), heel raises (Climie et al. (28), 2018; Evans et al. (29), 2019; Horiuchi et al. (32), 2022), and cycle ergometry (Eguchi et al. (44), 2013; Murtagh et al. (45), 2005).

### 3.4 Effects of sedentary interruption

**Figure 2:**
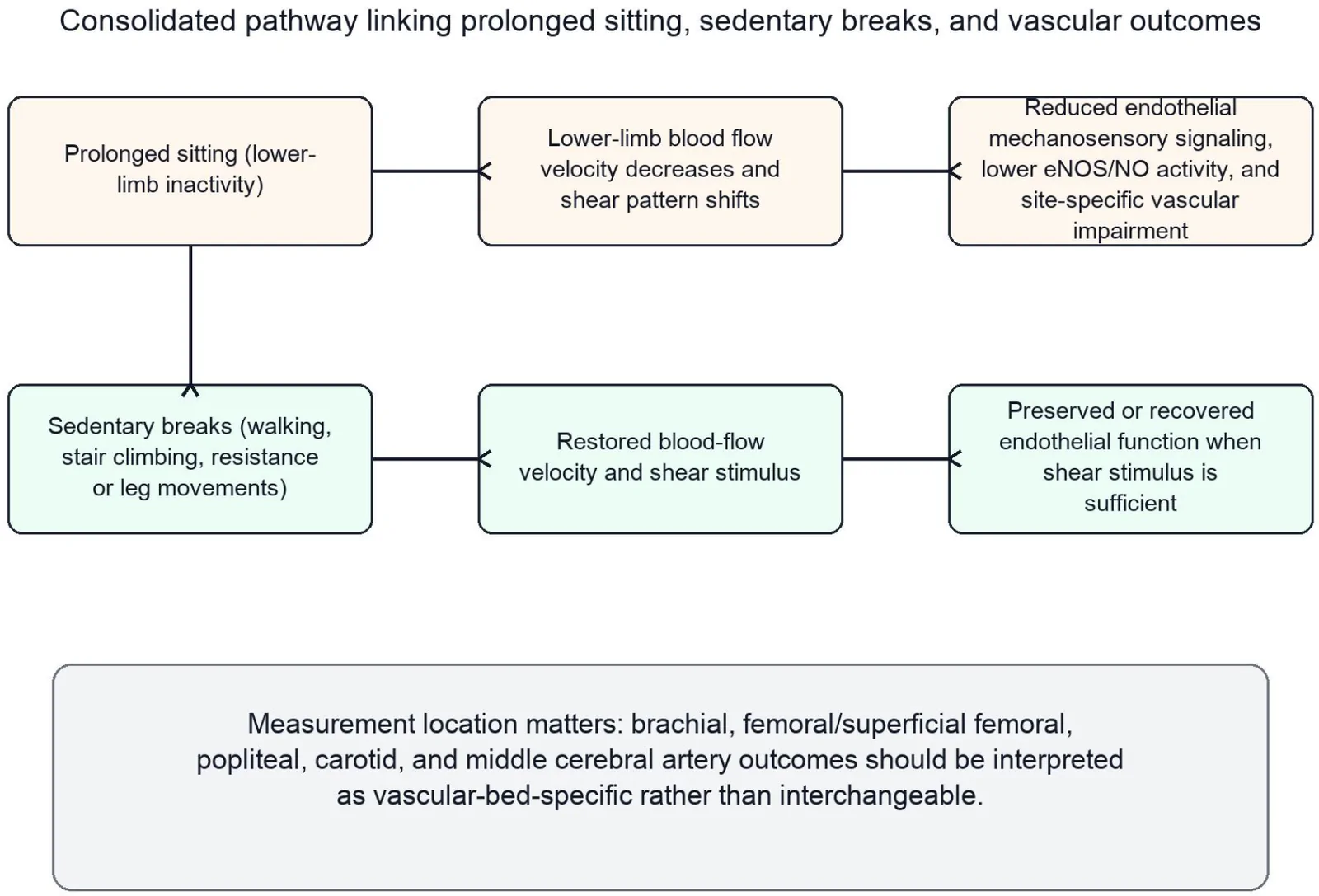
Consolidated mechanisms by which prolonged sitting and sedentary breaks affect blood flow velocity, shear rate, and FMD.

A consolidated mechanism diagram is provided in Figure 2; the results below focus on the vascular outcomes directly reported by the included studies.

#### 3.4.1 Effects of sedentary interruption on peripheral shear stress in adults

The reviewed studies assessed shear-rate outcomes in different vascular beds, most commonly lower-limb arteries. Therefore, shear-rate findings are reported below with the corresponding measurement location rather than being treated as a single interchangeable outcome.

Quantitative results for each study are summarized in Table 3. Among the 24 included studies, 12 reported outcomes related to shear rate or shear stress. Six studies reported improved shear-rate outcomes after sedentary breaks, including femoral or superficial femoral artery measures in Caldwell et al. (24), Carter et al. (26), and Taylor et al. (38, 39), popliteal artery measures in Morishima et al. (33) and Peddie et al. (35), and brachial artery shear-rate measures in Carter and Gladwell (27). In contrast, Chandran et al. (20) reported no significant improvement in carotid or superficial femoral artery hemodynamics after walking or stair-climbing breaks, while Kruse et al. (31) and Silva et al. (37) reported no significant improvement in the vascular outcomes assessed in their protocols.

The largest reported improvements were observed in studies using lower-limb dynamic or resistance-type interruptions. For example, Taylor et al. (38) reported increased superficial femoral artery shear rate after simple resistance activity in adults with type 2 diabetes, and Caldwell et al. (24) reported improved femoral artery shear patterns after hourly stair sprinting. These findings should be interpreted as lower-limb artery responses rather than as evidence for generalized improvement across all arterial beds.

#### 3.4.2 Effects of sedentary interruption on peripheral blood flow in adults

Blood-flow outcomes were also location-specific, with most peripheral measures obtained from lower-limb arteries. The following synthesis therefore distinguishes lower-limb blood-flow findings from cerebrovascular blood-flow velocity outcomes.

Among the 24 included studies, 11 reported outcomes related to peripheral blood flow. Eight studies demonstrated increased blood flow at the end of the intervention or during the sedentary-break condition, including femoral or superficial femoral artery measures in Caldwell et al. (24), Carter et al. (26), Climie et al. (28), and Taylor et al. (38, 39), popliteal artery measures in Morishima et al. (33) and Peddie et al. (35), and lower-limb vascular measures in Hartman et al. (30). Studies focused on cerebral blood flow velocity, including Carter et al. (25), Perdomo et al. (36), and Wheeler et al. (41), are reported separately because middle cerebral artery blood flow velocity reflects a different vascular bed.

Across the studies showing positive peripheral blood-flow responses, the reported benefits were most consistent in lower-limb arteries during interventions that repeatedly activated the lower-limb muscle pump, such as stair sprinting, simple resistance activity, walking breaks, or leg fidgeting. The three studies that did not observe significant peripheral blood-flow changes should be interpreted in relation to their specific protocols, participant characteristics, and measurement location, rather than as uniform null effects across all arteries.

Because reporting formats varied across studies, Table 3 provides the study-level blood-flow outcome together with the artery or measurement location. This location-specific presentation is intended to distinguish peripheral conduit-artery measures from cerebral blood flow velocity and other vascular indicators.

#### 3.4.3 Effects of sedentary interruption on peripheral flow-mediated dilation (FMD) in adults

Among the 24 included studies, 13 reported outcomes related to FMD. Five studies demonstrated significant protection or improvement of FMD at the end of the intervention, but the artery examined differed across studies: Hartman et al. (30) reported improved superficial femoral artery FMD and cerebral blood flow velocity in older adults at increased cardiovascular risk, Thosar et al. (40) reported protection of femoral artery FMD after walking breaks, Morishima et al. (33) reported preservation of popliteal artery FMD with leg fidgeting, Park et al. (34) reported preservation of brachial and popliteal artery FMD with active leg movement, and Taylor et al. (38) reported FMD-related benefits in the superficial femoral artery in adults with type 2 diabetes. These findings therefore indicate site-specific lower-limb or peripheral conduit-artery responses rather than a uniform FMD effect.

In contrast, eight studies (Caldwell et al. (24), Carter et al. (26), Carter and Gladwell (27), Climie et al. (28), Kruse et al. (31), Peddie et al. (35), Silva et al. (37), and Taylor et al. (39) reported no significant sedentary-break effect on FMD compared with uninterrupted sitting or the relevant control condition (*p* > 0.05 for all). These null findings included femoral/superficial femoral artery FMD, popliteal artery FMD, and brachial artery FMD, reinforcing that FMD findings should be interpreted according to the artery examined.

Because the Results section is restricted to the reviewed manuscripts, mechanistic explanations for these null findings are discussed in the Discussion rather than treated as direct study results.

#### 3.4.4 Effects of sedentary interruption on pulse wave parameters and cerebral blood flow velocity in adults

Among the 24 included studies, four reported outcomes related to pulse wave parameters, and three reported outcomes related to cerebral blood flow velocity. Horiuchi et al. (32) reported lower-limb arterial stiffness and microvascular perfusion outcomes after uninterrupted sitting and bodyweight exercise interruptions. Evans et al. (29) reported that heel-raise activity did not prevent the aortic stiffness response to acute prolonged sitting. Perdomo et al. (36) and Wheeler et al. (41) assessed cerebrovascular hemodynamics using middle cerebral artery blood flow velocity, whereas Carter et al. (25) examined cerebrovascular blood flow velocity responses to different walking-break frequencies.

For cerebral outcomes, the included studies should not be pooled with peripheral FMD or lower-limb blood-flow outcomes. Perdomo et al. (36) observed that alternating standing and sitting attenuated the midday decline in middle cerebral artery blood flow velocity compared with prolonged sitting. Wheeler et al. (41) reported that morning exercise combined with regular light-walking breaks improved daily cerebral hemodynamic profiles in older adults, while Carter et al. (25) showed that frequent short walking breaks were more effective than less frequent longer breaks for maintaining cerebral blood flow velocity during prolonged sitting.

#### 3.4.5 Effects of sedentary interruption on other cardiovascular disease (CVD) risk factors in adults

Among the 24 included studies, five reported outcomes related to other cardiovascular disease (CVD) risk factors rather than direct artery-specific endothelial function. These studies assessed outcomes such as cardiometabolic biomarkers, metabolic syndrome indicators, atherogenic indices, blood pressure, and lipid-related markers after accumulated or intermittent exercise protocols.

For example, Lim et al. (43) reported that intermittent exercise improved cardiovascular risk biomarkers in male night-shift workers, while Eguchi et al. (44), Murtagh et al. (45), and Murphy et al. (46) compared accumulated short bouts with longer continuous exercise bouts in working or sedentary adults. These studies were retained as secondary CVD-risk evidence, but they do not provide artery-specific FMD or shear-rate outcomes.

Overall, the CVD-risk-factor studies suggest that accumulated exercise can improve selected cardiometabolic outcomes, but these findings should be interpreted separately from the acute artery-specific endothelial function evidence summarized above.

## 4 Discussion

This systematic review found that breaking up prolonged sitting most consistently improved lower-limb hemodynamic outcomes, particularly blood flow and shear-rate responses, whereas effects on FMD were more heterogeneous. Importantly, the direction and magnitude of vascular responses differed according to the artery or vascular bed assessed. Lower-limb arteries such as the femoral/superficial femoral and popliteal arteries were most frequently examined, while several studies assessed brachial artery FMD or middle cerebral artery blood flow velocity. Therefore, the overall findings support sedentary breaks as a promising vascular-protection strategy, but the evidence should be interpreted in a measurement-location-specific manner.

The included studies reported outcome data in heterogeneous formats, with some reporting group means and standard deviations while others reported only *p*-values or percentage changes. This precluded uniform calculation of standardized effect sizes across all interventions. Methodological heterogeneity also arose from intervention frequency, duration, and intensity; participant characteristics; outcome timing; and measurement location. These factors help explain why improvements in blood flow or shear rate did not always translate into measurable FMD changes.

Sedentary-induced vascular impairment appears to be closely associated with sustained reductions in shear stress, but this mechanism is likely to vary by vascular bed. Lower-limb conduit arteries are directly affected by sitting posture, knee and hip flexion, venous pooling, and reduced skeletal muscle pump activity, whereas brachial artery FMD and middle cerebral artery blood flow velocity reflect different vascular beds and regulatory mechanisms. Consequently, a change in femoral, superficial femoral, or popliteal artery FMD cannot be assumed to have the same prognostic meaning as brachial artery FMD. This distinction is particularly important because the commonly cited association between FMD and future cardiovascular events is based on brachial artery FMD evidence (7, 8).

The effectiveness of different sedentary interruption strategies varies considerably. Dynamic lower-limb activities currently represent the most consistently effective approach for lower-limb blood-flow and shear-rate outcomes. Walking, stair sprinting, leg fidgeting, and simple resistance activity can increase local shear stimuli in femoral/superficial femoral or popliteal arteries, but their effects on FMD are not uniformly significant across studies. Taylor et al. (39), for example, examined superficial femoral artery responses in women with polycystic ovary syndrome, whereas Climie et al. (28) measured femoral artery function and brachial FMD in overweight/obese adults. These findings suggest that intervention dose and the vascular bed examined jointly influence whether an endothelial benefit is detected.

With respect to cerebral blood flow, Wheeler et al. (41) observed that morning exercise combined with walking-based sedentary interruptions improved daily cerebral hemodynamic profiles in older adults, whereas uninterrupted sitting led to sustained reductions in middle cerebral artery blood flow velocity. Carter et al. (25) further showed that high-frequency, short-duration walking counteracted the decline in cerebral perfusion more effectively than lower-frequency walking breaks. These cerebral blood flow velocity findings should be interpreted separately from peripheral FMD findings because they reflect different vascular physiology and measurement methods.

Localized active or passive movements may also have potential value, but their effects appear to depend on the vascular bed. Morishima et al. (33) demonstrated that intermittent leg fidgeting protected popliteal artery endothelial function by increasing local shear rate. Park et al. (34) further showed that active leg movement protected macrovascular endothelial function, whereas passive movement was more effective for microvascular outcomes. These observations highlight mechanistic differences between microvascular and macrovascular adaptations and reinforce the need to report the artery or measurement location in future studies.

Importantly, responses to sedentary interruption vary across populations. Healthy young adults generally exhibit preserved baseline endothelial function and may show smaller intervention effects, whereas individuals with obesity, type 2 diabetes, polycystic ovary syndrome, hypertension/prehypertension, or older age may have lower baseline endothelial function and altered responsiveness to shear stimuli. However, greater baseline risk does not guarantee FMD improvement, particularly when the measured artery, interruption intensity, or observation window differs across studies. These findings underscore the necessity of population-specific and artery-specific intervention strategies.

Despite confirming multiple benefits of sedentary interruption, this review also highlights important limitations of the existing evidence. Most studies were short-term, acute trials, with a lack of long-term follow-up data. Hartman et al. (30) suggested that sustained reductions in sedentary behavior combined with timely interruptions may yield cumulative improvements in cerebral blood flow and vascular function; however, the small sample size limits the generalizability of these findings. Moreover, the majority of studies were conducted in controlled laboratory settings, and intervention adherence and long-term feasibility have yet to be systematically evaluated in real-world occupational or daily-life environments (47–49).

Based on the findings of this review, prolonged uninterrupted sitting should be minimized in daily life and workplace settings. Frequent dynamic lower-limb activities performed every 20–30 min for 2–3 min, such as walking, heel raises, semi-squats, or stair climbing, appear most likely to enhance lower-limb blood flow and shear-rate profiles. However, recommendations regarding FMD should specify the measurement location, because brachial, femoral/superficial femoral, and popliteal artery FMD responses are not interchangeable.

In conclusion, interrupting sedentary behavior represents a low-cost and feasible strategy for protecting vascular health, especially by improving lower-limb hemodynamic responses during prolonged sitting. Future studies should standardize reporting of artery-specific outcomes, intervention dose, timing of measurement, and participant risk profile so that vascular-bed-specific effects can be compared more accurately.

## 5 Conclusions and future directions

### 5.1 Conclusions

This systematic review demonstrates that prolonged sitting can rapidly impair vascular function, whereas appropriately timed activity breaks may improve blood flow and shear-rate outcomes, particularly in lower-limb arteries. Performing 2–3 min of dynamic lower-limb activity or simple resistance exercise every 20–30 min appears to be a practical strategy for improving peripheral hemodynamics, although FMD effects remain artery-specific and should be interpreted separately for brachial, femoral/superficial femoral, and popliteal artery measurements. Sedentary interruption may also confer potential benefits for cerebral blood flow regulation, especially in older adults or individuals with metabolic risk, but the optimal strategy may vary according to age, health status, baseline vascular function, and measurement location (26, 38, 39).

### 5.2 Future directions

Future research should further advance this field in several key areas. First, systematic efforts are needed to identify the optimal combinations of frequency, duration, and exercise intensity across different sedentary interruption strategies. Second, future studies should stratify participants by age, metabolic risk, baseline vascular function, and medication status. Third, artery-specific reporting should be standardized: studies should clearly identify whether outcomes are measured in the brachial, femoral/superficial femoral, popliteal, carotid, or cerebral circulation and avoid extrapolating FMD findings across vascular beds. Finally, long-term follow-up and real-world occupational implementation studies are required to determine whether sedentary interruption produces sustained arterial benefits and reduces cardiovascular event risk.

## Data Availability

The original contributions presented in the study are included in the article/Supplementary Material, further inquiries can be directed to the corresponding author.
